# Enucleation and Evisceration in the Palestinian Territories

**DOI:** 10.4103/0974-9233.80708

**Published:** 2011

**Authors:** Tiarnan D. L. Keenan, Nicholas J. Sargent

**Affiliations:** 1 University of Manchester and the Manchester Royal Eye Hospital, Manchester, United Kingdom; 2 St. John Eye Hospital, Jerusalem, Palestinian Territories

**Keywords:** Crowd Control, Enucleation, Evisceration, Ocular Trauma, Rubber Bullets

## Abstract

**Purpose:**

To examine the demographics and indications in patients undergoing eye removal at St. John Eye Hospital (SJEH) in Jerusalem, the largest single provider of ophthalmic care in the Palestinian Territories.

**Materials and Methods:**

In this retrospective study, medical records were reviewed for patients undergoing enucleation or evisceration at SJEH from November 2004 to March 2007. Calculation of percentage, mean and median was performed for the demographics, and indications for enucleation and evisceration.

**Results:**

Thirty-three eyes of 32 patients were removed during the period under study. Twelve enucleations and 21 eviscerations were performed. Mean age was 39 years, and 19 patients were male. Indications included severe trauma (8 eyes), painful blind eye with (5 eyes) or without (9 eyes) infection, and ophthalmic neoplasm (3 eyes).

**Conclusion:**

The incidence of surgical eye removal at SJEH from 2004 to 2007 was around one patient per month for a population over three million. This rate appears far lower than those reported in previous studies of similar Palestinian populations. Prompt access to medical care for Palestinians is required to mitigate ophthalmic morbidity. Approximately half of the cases were caused by severe trauma or infection, with rubber bullet injuries responsible for 20% of the traumatic cases.

## INTRODUCTION

Enucleation or evisceration may be required for advanced ophthalmic disease, particularly where the visual prognosis for the eye is extremely poor. Enucleation involves the surgical removal of the entire globe, including the sclera, and is achieved by division of the extraocular muscles and optic nerve. Evisceration refers to removal of the intraocular contents, leaving the sclera and optic nerve intact. These surgical approaches may be required in the context of overwhelming infection, severe trauma, or ophthalmic neoplasm, and may also be appropriate in the presence of a painful blind eye or for cosmetic indications.

The incidence, demographic features, and indications for enucleation and evisceration have not been reported in the recent literature for the Palestinian Territories.^1^ The demographics and indications for enucleation are likely to differ significantly from those in Europe or North America due to difficulties with prompt access to medical treatment,^2^ increased prevalence of some ophthalmic conditions^3^ and higher incidence of eye trauma through work-related injuries and armed conflict.^4^–^5^

St. John Eye Hospital, Jerusalem (SJEH), is the largest single provider of ophthalmic care in the Palestinian Territories, which comprise East Jerusalem, the West Bank, and the Gaza Strip. The population of the Palestinian Territories in 2007 was estimated at 3,761,600, including 1,416,500 in the Gaza Strip.^6^,^7^ Palestinians with a West Bank or Gaza identity card can enter Jerusalem via military checkpoints only with a specific permit; these permits may be denied or delayed such that access to secondary and tertiary referral centers in East Jerusalem is restricted.^2^,^8^–^10^ The SJEH group also runs the Gaza Clinic, a small ophthalmic hospital in the Gaza Strip. The Gaza Clinic does perform day surgery but during the study period was unable to perform any evisceration or enucleation operations, as there was no access to the facilities and consumables required for general anesthesia. In this context, SJEH was the largest single provider of enucleation or evisceration services for a population of 3,761,600 people over the course of the study period.

## MATERIALS AND METHODS

The medical records were reviewed for all patients undergoing enucleation or evisceration at SJEH (identified from surgical logbooks) from November 2004 to March 2007. Data collected included age (at time of surgery), sex, eye affected, operative details, surgical indication, and underlying disease or cause of trauma.

## RESULTS

Thirty-three eyes of 32 patients underwent enucleation or evisceration at SJEH from November 2004 to March 2007. Twenty-one patients (21 eyes) underwent evisceration, 10 patients (11 eyes) underwent enucleation, and 1 patient underwent unilateral enucleation and excision of an orbital mass. Nineteen out of the 32 patients were male, and the left eye was removed in 21 out of 33 eyes.

The number of eyes removed subdivided by surgical indication is shown in Table 1, and by underlying disease in Table 2. The most common underlying problems leading to removal were trauma (8 eyes of 8 patients) and intraocular infection (6 eyes of 6 patients).

**Table 1:** Indication for enucleation or evisceration from November 2004 to March 2007 at St John Eye Hospital, Jerusalem

Indication	Number	Percentage	Male: female ratio	Age: mean, median, range
Severe trauma	8	24	6:2	35; 26; 8-70
Painful blind eye without intraocular infection	9	27	5:4	41; 28; 18-74
Painful blind eye with intraocular infection	5	15	3:2	57; 75; 1-79
Neoplasm	3	9	0:3	40; 3; 0-76
Cosmesis	2	6	1:1	20; 20; 18-22
Other / unknown	6	18	5:1	Unknown
Total	33	100	20:13	39; 24; 0-79

**Table 2:** Type of ocular disease in eyes that underwent enucleation or evisceration from November 2004 to March 2007 at St John Eye Hospital, Jerusalem

Ocular disease	Number	Percentage	Male: female ratio	Age: mean, median, range
Severe trauma	8	24	6:2	35; 26; 8-70
Intraocular infection	6	18	3:3	57; 75; 1-79
Glaucoma	4	12	2:2	34; 21; 20-74
Neoplasm	3	9	0:3	40; 3; 0-76
Phthisis bulbi	3	9	2:1	19; 18; 18-21
Vitreo-retinal disease	1	3	0:1	70; 70; 70
Other / unknown	8	24	7:1	54; 54; 35-72
Total	33	100	20:13	39; 24; 0-79

The age of patients (where recorded in the notes) is shown in Tables 1–3. Mean age of all patients was 39 years (median, 24 years and modal decade, seventies), but age showed different distributions according to diagnostic indication. The median age of 8 patients (6 males and 2 females) undergoing surgery following trauma was 26 years, but 75 years for 6 patients (3 males and 3 females) undergoing surgery for intraocular infection. Three females underwent surgery for neoplasm, a 3-month-old baby for unilateral retinoblastoma, a 3-year-old child for unilateral retinoblastoma, and a 76-year-old woman for orbital meningioma.

**Table 3:** Age of patients who underwent enucleation or evisceration from November 2004 to March 2007 at St John Eye Hospital, Jerusalem

Age of patient (years)	0-9	10-19	20-29	30-39	40-49	50-59	60-69	70-79	All
All patients	4	3	6	1	1	0	1	8	24
Severe trauma	1	1	2	0	1	0	1	1	7
Intraocular infection	1	0	0	0	0	0	0	3	4
Neoplasm	2	0	0	0	0	0	0	1	3

Data on age were not available for all patients

One 22-year-old female underwent evisceration following a rubber bullet injury, although the bullet had already removed the ocular contents. An 18-year-old male underwent enucleation of a blind phthisical eye for cosmetic indications; the original injury 5 years earlier involved a rubber bullet, which penetrated the orbit and lodged in the ethmoidal sinus. Mechanisms of injury in the other cases included injuries with stones (2 males), an object thrown from a fly-wheel (1 male at work), the edge of a table (elderly female at home), a wooden stick (young boy playing) and a metal spring (young boy playing). Five of the six patients undergoing surgery for intraocular infection had severe corneal disease as the cause, while the remaining patients were diagnosed with endophthalmitis following complicated cataract surgery at another hospital. One 20-year-old male underwent bilateral sequential enucleation with primary orbital implants, with a silicone ball implant in one eye and a polyethylene Medpor implant (Porex Surgical, Inc, College Park, GA, USA) in the fellow eye, for blind painful eyes with end-stage secondary glaucoma following surgery many years earlier for congenital cataract.

## DISCUSSION

The incidence of surgical eye removal in the Palestinian Territories at SJEH from November 2004 to March 2007 was approximately one patient per month for a population of over three million. Trauma or severe ocular infection was the cause of approximately half the cases requiring surgical removal. The removal rate in our study appears far lower than the 409 eyes reported by Batten^1^ at SJEH from 1965 to 1969. However, SJEH was relocated in 1967 from Jordan to East Jerusalem, and referral patterns changed significantly. Batten^1^ also found that enucleation was performed more commonly than evisceration, in contrast to the current study. The male preponderance was similar in our study to that of Batten’s study,^1^ at around 3:2, although Batten’s series^1^ showed a fairly even distribution of case numbers over all age groups. Painful blind eye accounted for 25% of enucleations, corneal disease for 25% and trauma for 18% in Batten’s study,^1^ which is similar to the indications in our study.

The rate of surgical eye removal found in this study also appears far lower than that reported by Jaouni and O’Shea^4^ during the First Intifada (1987-1993). However, the relative distribution of indications for eye removal was dramatically biased toward ocular trauma at this time. During the 6-year study period reported by Jaouni and O’Shea,^4^ 154 eye injuries caused by rubber or plastic bullets were treated at SJEH. In particular, 86 enucleations were performed, of which 78 (91%) were for rubber or plastic bullet injuries.

The relative distribution of indications for enucleation and evisceration were similar in this study to those reported by Gaton and colleagues^11^ at a large ophthalmology department in Tel Aviv, Israel, from 1981 to 2007. Gaton and colleagues^11^ reported trauma in 32.6%, blind painful eye in 27.6%, endophthalmitis in 27.2%, and neoplasm in 12.6%. However, patients undergoing surgery for intraocular infection in our study generally had severe corneal disease rather than postoperative endophthalmitis as the underlying cause. This is likely explained by delayed presentation and poor access to ophthalmology services in the Palestinian Territories,^2^ and the relatively low rate of cataract surgery.^3^

In conclusion, we emphasize the critical importance of prompt access to appropriate medical care; this may involve urgent referral with prioritized access to SJEH, especially for acute trauma, severe infection or suspected neoplasm (particularly children with possible retinoblastoma). In addition, it is likely that many patients in the Gaza Strip require surgery but have been unable to attend, and may be living with ocular pain or disfigurement without useful vision. We question the use of rubber bullets for crowd control,^12^ and recommend appropriate eye protection at work, supervision of children at play, and improved access and financial support for patients requiring treatment for eye disorders, such as glaucoma and corneal disease.
